# Micrornas in autoinflammation and autoimmunity

**DOI:** 10.1186/1546-0096-12-S1-I14

**Published:** 2014-09-17

**Authors:** Florence Apparailly

**Affiliations:** 1 U844, INSERM, Montpellier, France

## 

Micro(mi)RNAs are small non-coding RNAs that play critical roles in physiological networks by regulating genetic programs. They are conserved from worms to mammals and function as negative regulators of protein-encoding gene expression. Research on the role of miRNAs in pathophysiological conditions is very active since 10 years and several works evidenced that miRNAs play a key role in the regulation of immunological functions and the prevention of autoimmunity. I will discuss the involvement of miRNAs in the regulation of innate and adaptive immune functions and in the development of autoimmune disease. Focusing on the role of few miRNAs, I will emphasize the intertwined relationships between tissue homeostasis and immunity, and on how studying miRNAs in autoimmunity and immune-mediated inflammatory disorders will shed light on pathological processes and help identifying novel drug candidates and biomarkers.

## Disclosure of interest

None declared.

